# Vascular implants – new aspects for in situ tissue engineering

**DOI:** 10.1002/elsc.202100100

**Published:** 2022-01-07

**Authors:** Cornelia Blume, Xenia Kraus, Sebastian Heene, Sebastian Loewner, Nils Stanislawski, Fabian Cholewa, Holger Blume

**Affiliations:** ^1^ Institute for Technical Chemistry Leibniz University Hannover Hannover Germany; ^2^ Institute for Microelectronic Systems Leibniz University Hannover Hannover Germany

**Keywords:** bioreactor design, tissue engineering, vascular implants, 3D printing

## Abstract

Conventional synthetic vascular grafts require ongoing anticoagulation, and autologous venous grafts are often not available in elderly patients. This review highlights the development of bioartificial vessels replacing brain‐dead donor‐ or animal‐deriving vessels with ongoing immune reactivity. The vision for such bio‐hybrids exists in a combination of biodegradable scaffolds and seeding with immune‐neutral cells, and here different cells sources such as autologous progenitor cells or stem cells are relevant. This kind of in situ tissue engineering depends on a suitable bioreactor system with elaborate monitoring systems, three‐dimensional (3D) visualization and a potential of cell conditioning into the direction of the targeted vascular cell phenotype. Necessary bioreactor tools for dynamic and pulsatile cultivation are described. In addition, a concept for design of vasa vasorum is outlined, that is needed for sustainable nutrition of the wall structure in large caliber vessels. For scaffold design and cell adhesion additives, different materials and technologies are discussed. 3D printing is introduced as a relatively new field with promising prospects, for example, to create complex geometries or micro‐structured surfaces for optimal cell adhesion and ingrowth in a standardized and custom designed procedure. Summarizing, a bio‐hybrid vascular prosthesis from a controlled biotechnological process is thus coming more and more into view. It has the potential to withstand strict approval requirements applied for advanced therapy medicinal products.

**Abbreviations**
iPS: inducible pluripotent stem cells
ECM: extracellular matrix
TEVG: tissue‐engineered vascular grafts

## 1 INTRODUCTION

Cardiovascular diseases due to atherosclerosis of coronary or peripheral arteries list as the number one cause of death globally by the World Health Organization. Substitution of blood vessels is required when the vasculature is affected by severe atherosclerosis or even stenosis. Besides synthetic vascular grafts with the necessity of ongoing anti‐coagulative treatment, autologous venous substitutes are often not available in the elderly, and allogeneic vascular grafts from brain‐dead donors require con‐comitant immunosuppression. Up to now, here the use of autologous vessels represents the gold standard for vessels with a diameter <6 mm, but bio‐artificial alternatives for narrow‐caliber vessels are urgently needed. Such bioartificial grafts ‐ manufactured with autologous cells and a suitable vascular prosthesis material are being developed. The prospective approach is to bypass diseased vessels with an up to three‐cell‐layer‐containing tubular scaffold as wall structure [1]. In this context, 3D printing plays a promising role [2].

The principle of developing a tissue‐engineered vascular graft (TEVG) includes several steps: suitable autologous vascular cells such as endothelial progenitor cells or vascular smooth muscle‐outgrowth cells could be isolated, for example, from a patient's peripheral blood. Alternatively, inducible pluripotent stem cells (iPS) or allogenic adult stem cells out of bone marrow or liposections or other niches of the human body are likely to be prepared and consequently differentiated to vascular cells. Human iPS are currently being explored for this application [3], since iPS represent only a small risk for immunogenicity. Attempts have also been made with more immune reactive xenogeneic cells. After extra‐corporal cell expansion, cells are seeded on suitable vascular scaffold structures. Here xenogeneic decellularized vessels were tested, and in addition, different biocompatible materials were evaluated as possible vascular scaffolds using methods such as electrospinning or currently also 3D printing. For increased cell adhesion, scaffolds were functionalized by coating with natural polymers such as human fibrin and others. The bio‐artificial graft can either be immediately implanted for maturation within the given milieu of a living organism, or it can be cultivated over a period, ranging up to several weeks in a specialized bioreactor. Bioreactors are a key technology for the in vitro cultivation of biological substitutes [4]. These reactors support the generation of functional implants by providing efficient internal and external mass transport to and from the cells while maintaining the required environmental conditions (e.g., temperature, pressure, shear stress, oxygen and carbon dioxide saturation, pH, metabolites) for cell growth. Throughout the entire process, this environment must be kept sterile and the bioreactor should remain sealed after initial assembly. Ideally, all featured sensors and actuators should have no contact to the culture medium to ensure a sterile, simple, and wasteless setup. Continuous observation needs to provide data on the growth process, internal environment, and the geometric structure of the vascular graft inside the bioreactor to allow for automated processing [5]. Automatization and parallelization of cultivation processes improves research and process development as well as subsequent production runs. This review will highlight all aspects of TEVG development and outline, which technical innovations will drive the progress in the design of TEVGs in the near future.

**PRACTICAL APPLICATION**

This review highlights the biotechnical issues and clarifies, what are the necessary prerequisites to cultivate a vascular graft as replacement vessels for clinical use. In an elderly society, there is a huge need of new bioartificial vessels, since patients often do not have enough autologous vascular substitutes. The article discusses different scaffold and hydrogel materials, cell sources with their risks and advantages as well as bioreactor technologies including suitable cultivation monitoring tools. The significance of the extra‐corporal cultivation process for such grafts is explained, which is the basis for physiological vascular maturation. The importance of 3D printing for vascular scaffold design is outlined. Among the diverse concepts, bio‐hybrid vascular structures cultured in a perfusion bioreactor appear as a realistic possibility for near clinical use.

## 2 WHICH INNOVATIONS ARE NEEDED AND WHY?

### 2.1 Non‐immunogenic cells

Since xenogeneic cells impose a risk of inter‐species rejection, firstly human allogenic cells were tested for TEVGs. The problem of extra‐corporal expansion of these cells was remarkably solved by Poh et al., who developed a method to extend the lifespan of human endothelial cells isolated from senior donors. The group used genetic manipulation to achieve increased telomerase expression and growth [5]. Lawson et al. also used this concept with allogenic cells, and seeded poly(glycolic acid) (PGA) scaffolds with these gene‐manipulated smooth muscle cells (SMCs). TEVGs were dynamically cultured and decellularized, and the resulting purely extracellular matrix (ECM) structures were implanted as dialysis access [6]. As much as 33% of the shunts showed a so‐called primary patency in patients over 12 months without intervention, but in 95% of the cases, patients received interventions such as thrombectomy, angioplasty and others. Although this is a highly complex and intelligent approach, residual allogenic cell material may possibly have contributed to the high rate of vascular intervention procedures that were necessary to hold the artificial vessels open. As xenogeneic and even allogenic human cells thus impose a risk of rejection, other and immune‐neutral cell sources are needed. New concepts currently deal with the use of iPS [3] and aim to optimize differentiation processes of adult stem cells with a presumably lower immunogenic profile than mature cells. Such stem cell differentiation to vascular cells for TEVGs was already tested in an animal model as an example protocol for human stem cells [7]. In the work of Gui et al., bovine stem cells differentiated into smooth‐vascular muscle‐like cells and were successfully expanded before seeding on polymers. The resulting vascular grafts unfortunately revealed an inferior biomechanical stability in nude rats, possibly due to the fact, that the differentiation process did not reach the quality of end‐differentiated contractile SMCs. Besides the research on stem cells, other concepts are being developed for autologous cells in high cell numbers [8].

### 2.2 Vascular grafts with sufficient stability for patients

In the last 5 years, new aspects have emerged in the field of developing new (bio‐) hybrid vascular prostheses. Concepts that have proven themselves to the point of use in patients now often rely on bioresorbable synthetic scaffolds. In the past, Niklasson and Lange have already set an important model approach for new developments in this field with a synthetic scaffold for a small caliber poly(glycolic acid) (PGA) vessel with bovine cells at that time [9]. Referring to this approach, the group of Shinoka [10] developed new vascular grafts from polylactid acid (PLA)/PGA scaffolds, which were seeded with human bone marrow‐derived vascular muscle cells. Implanted in 25 patients, these vessels showed a durability over 11 years before approval by the FDA. A follow‐up study in children, started in 2019, is still ongoing [11] and revealed only one patient with thrombosis in the early phase, which underlines the meaning of human cells used in this approach. However, these grafts were affected by stenosis in almost one‐third of cases due to suboptimal biomechanical properties. In contrast, sustained anatomical and functional stability was achieved for a TEVG based upon a synthetic scaffold in children, which was cellularized with autologous bone marrow mononuclear cells. These electrospinned polyester vessels were implanted and followed over 12 months [12]. The polyester used was obtained by chain‐extending poly‐caprolactone (PCL) with a self‐complementary acting 2‐ureido‐5[1H]‐pyrimidone. The latter one formed quadruple hydrogen bonding and thus supported biomechanical properties. Others tested a cell‐free approach denoted as “Vascudyne®” as hemodialysis shunt [13]. Here neonatal human dermal allogenic fibroblasts were entrapped in bovine fibrin gel and the resulting tubes were subsequently decellularized. These “off‐the‐shelf”‐vessels represent a complete biotechnological production (non‐living). Tested in a baboon model under daily antiplatelet therapy, these shunts showed 3‐ to 6‐months primary patency rates of 83% and 60%, respectively. At explant, the patented grafts were recellularized, not calcified and showed a stable biomechanical stability.

### 2.3 Dynamic cultivation in a bioreactor

The principle of an extracellular cultivation period inside a suitable bioreactor can be referred to as “in situ tissue engineering.” This concept was followed in parallel to approaches suggesting the use of a so‐called “living bioreactor,” that means the spontaneous implantation of the artificial vascular graft into a living organism for further seeding and integration into the vascular system of the recipient. As an example for this living bioreactor, Dahan et al. could successfully implant vascular grafts directly into an animal model, where spontaneous colonization with endothelial cells occurred in vivo [14]. Which concept is favorable, is a matter of constant debate and may depend on the vision for the later surgical application. While the in vivo, colonizing‐concept supports emergency surgeries where prepared vessels are immediately available, in vitro created grafts represent highly complex functional implants that are only suitable for a plannable surgery.

To optimize the seeded surface of the scaffolds and avoid thromboembolic complications of these artificial vessels, various surface modifications for the synthetic materials have been tested and the principle of dynamic cultivation of colonized scaffolds prior to implantation was found to be advantageous [9]. Luo et al. [15] used human iPS on PGA scaffolds, and in his approach, the bio‐hybrid vessel was stimulated by stretching in a bioreactor system, which had an augmenting effect on collagen synthesis and SMC proliferation. Dynamic cultivation therefore is important not only to induce anti‐thrombogenic markers [8], but makes a difference in terms of biomechanical stability.

### 2.4 Summary

A synthetic tubular scaffold and non‐immunogenic endothelial and vascular smooth‐muscle (‐like) cells can thus be marked as essential prerequisites for a standardized generation of bio‐hybrid vascular prostheses. Such bio‐hybrid vessels should undergo dynamic cultivation and conditioning in an intelligent bioreactor, and this total concept represents an attractive approach with high potential to reach clinical usability.

## 3 TECHNICAL TRENDS IN BIOREACTOR DESIGN

The design of an intelligent bioreactor system must feature various sensory elements, a robust system setup and the possibility to reuse the bioreactor containment for several cultivation processes of TEVGs. Different kinds of TEVG bioreactor setups were presented over the past decades, with perfusion bioreactors as the predominantly‐used model [16, 17, 18, 19, 20] Features such as specifically compact [18] and cost‐efficient [20] designs promote high scalability.

Different papers emphasize such specific important bioreactor features. For example, the bioreactor of Huang et al. [21] offers the application of uni‐ or bi‐axial stresses on cultivating TEVGs using a linear motor. The presented bioreactor allows cultivating three vessels in parallel. Baba et al. [18] present a combined automated culture system for tubular structure assembly with enough space for dynamic stretching. The group achieved this by designing an in vivo‐like culture chamber made of polydimethylsiloxane (PDMS). Online monitoring of the cultivation process is possible by an integrated digital microscope. The aspect of dynamic conditioning in the bioreactor is also addressed by Wolf et al. [20]. Here, a disposable system featuring low costs for the disposable components is presented. The system offers a high degree of flexibility with a relatively wide range of adjustable parameters like, for example, the flow, pressure and pulse rate and pulse frequency parameters. The included micro‐centrifugal pump was capable of creating venous and arterial pressure levels. Because of this flexibility, the system is denoted as VascuTrainer.

**FIGURE 1:**
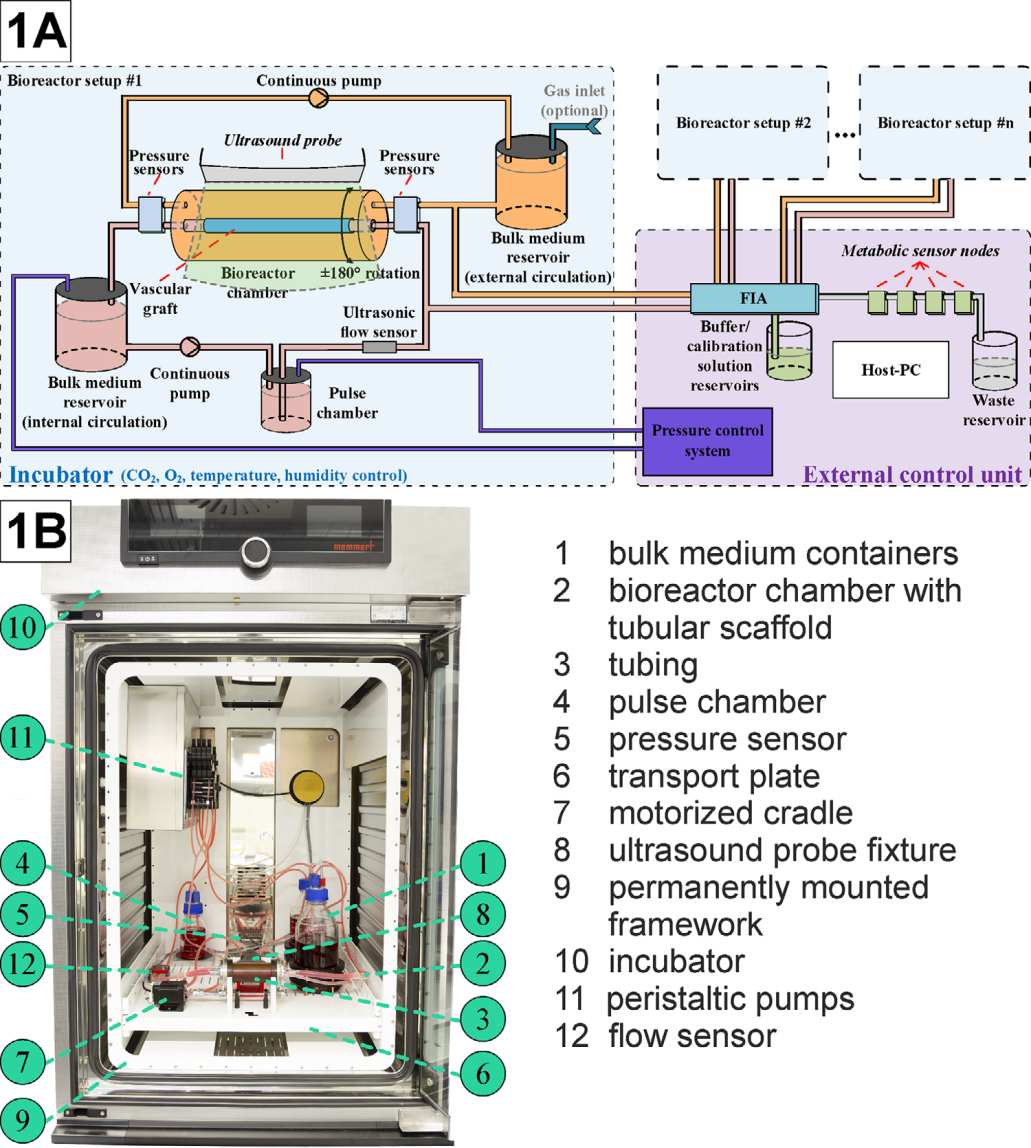
Bioreactor setup. (A) Schematic of a bioreactor setup for in vitro cultivation of a TEVG with external control unit for parallelization of cultivation processes; (B) Demonstration of assembled Bioreactor Setup [24]

Another comprehensive cultivation process for TEVGs (featuring, e.g., automated concentration measurement of glucose or lactate, online 3D visualization, shear stress analysis, etc.) has been elaborated and improved over several years at the Leibniz University of Hannover in [22, 23, 24]. This rotational‐perfusion bioreactor system comprises an automated monitoring and control of physiological conditions, and an online 3D visualization and geometrical analysis system. A schematic of this in vitro bioreactor setup is depicted in Figure 1.

The central element of the setup is the bioreactor chamber constructed out of polyetherimide, a high performance plastic material resistant to temperatures above 130°C, therefore suitable for heat sterilization. Furthermore, the material allows for the application of ultrasound imaging. The bioreactor has a tubular shape with an inner diameter of 3 cm. Depending on the required length of the implant, different lengths of the bioreactor up to 14 cm are possible. The tubular scaffold seeded with autologous cells is fastened centrally between the two end covers of the bioreactor chamber. Each end cover has two connectors for liquid medium supply. The diameter and lengths of the connectors vary with regard to the scaffold dimensions. The inner medium circulation (red) leads through the vascular graft and simulates the flow of blood. The outer circulation (orange) is used to fill the space surrounding the graft with liquid medium, simulating the interstitial space.

The bioreactor is mounted on a motorized cradle driven by a stepper motor to rotate the graft ± 180 ° and avoid twisting the connected flexible tubes. Combining the characteristics of rotational and perfusion bioreactors into one system results in (i) enhanced mass transfer not only at the construct periphery but also within the internal pores of the tissue and (ii) prevention of gravitational effects on the graft which might lead to asymmetric cell growth during cultivation.

### 3.1 Monitoring and control of physical conditions

To ensure the quality of a TEVG, an automated monitoring and control system is necessary. The system presented in Figure 1 includes a central control unit (Host‐PC) collecting data from all sensors and providing the required signals for all actuators. Two continuously running peristaltic pumps transport the medium inside the corresponding circulation and its bulk medium reservoir. Metabolic waste products are locally removed from the small medium volume enclosed in the graft and diluted in the circulating medium and bulk medium reservoirs. Pressure is monitored close to the bioreactor chamber both at the inlet and outlet of the inner and outer medium cycle. This information can be used for online shear stress simulation estimating wall‐near shear stress (e.g., using COMSOL Multiphysics). This is important because pressure is not only used to meet stability criteria, but also as a differentiation factor for stem cells or progenitor cells later during the cultivation process.

Pressure conditions as in the human circulation (up to ∼160 mbar in adults, up to ∼60 mbar in a fetal circulation at the time of embryogenesis and initial vessel formation) represent a differentiation signal for maturing vascular cells. However, due to the blood flow and regarding the Hagen‐Poiseuille flow laws applicable to physiological conditions, pressures significantly lowered compared to the system pressure act on the inner skin of the vessels in the range of maximum 50 dynes/cm^2^. This is due to the formation of a local “lubrication chamber” in the flow process of the blood [25]. Shear stress up to 25 dynes/cm^2^ and over several days was successfully established in suitable shear stress mini‐compartments, leading to enhanced expression or activation of cell differentiation markers. Pressure here was applied intermittently or uniformly to support the maturation of differentiated endothelial cells (e.g., indicated by vascular endothelial growth factor receptor‐2, von‐Willebrand‐factor or vascular endothelial‐cadherin), or SMCs (e.g., α‐smooth muscle actin or smooth muscle myosin heavy chain) [26, 27]. In addition, cells developed an anti‐thrombogenic gene profile [8]. Besides conditioning cell phenotypes, bioreactors were used to train and test stability of the matured vascular graft, and here a pressure load up to 2000 mbar and a suture tear strength of up to 3 mm/min have been realized in comparable systems [28].

In the system presented above (Figure 1), the physiological pressure profile and flow rate inside the vascular graft needed can be established by variation of pressure on the medium in an additional pulse chamber, placed between the pump for the internal circulation and the bioreactor chamber. At the beginning of the cultivation process, a low and constant pressure gradient is applied to the inner medium circulation to realize a shear stress of maximally 0.5 dynes/cm^2^ on the inner wall of the cultivated TEVG, which is required for a optimal cell adhesion to the scaffold. The baseline pressure is altered by applying pressure on the medium in the bulk medium container of the internal circulation. A non‐invasive ultrasonic flow sensor can be placed upstream of the vascular graft to assess the flow rate and to detect air bubbles. The rotating bioreactor chamber, bulk medium container, tubing, and all attached actuators and sensors are placed inside an incubator to provide temperature and humidity conditions appropriate for the cultivation process. Electronics located inside the incubator are sealed against the harsh conditions. All electrical components placed inside the incubator are specifically designed to minimize power dissipation to reduce the risk of overheating the well‐insulated incubator and the components placed inside.

### 3.2 Monitoring and control of metabolic conditions

To realize a close cultivation control, the exemplarily presented bioreactor system includes enzyme‐based electrochemical biosensors to monitor the glucose and lactate concentration of the media [24]. These disposable sensors must be periodically calibrated and intermittently flushed with saline buffer solutions to prolong the sensors’ lifetime. During long‐term cultivation, timely exchange of the medium can thus be ensured. Hydrogen peroxide created during enzymatic measurement of glucose and lactate has to be removed as possible cell toxin and disturbing factor for sensor calibration. Therefore a sample based flow injection analysis (FIA) setup located outside the incubator was added to the system.

Ambient oxygen and carbon dioxide concentrations can be controlled and monitored by the incubator. Gas‐permeable tubing allows gas exchange with the medium. Physiological conditions for either one of the circulation cycles can be independently controlled by deploying a separate gas inlet for the conditioning of the medium and non‐permeable tubing for the respective medium circulation.

pH and partial pressures of both oxygen (pO_2_) and carbon dioxide (pCO_2_) in the medium can be controlled online by fluorescent analyte‐sensitive sensor spots. Since only small changes of these parameters are expected, a low frequency sampling rate is needed and the sensors can be located outside the medium circulation as part of the established FIA. This also results in decreased costs of these disposables since both medium circulations can be assessed with only one (non‐sterile) sensor for each parameter. All sensors that are part of the FIA system can be used to monitor multiple bioreactor setups, thus decreasing overall cost and setup time.

### 3.3 Monitoring by 3D visualization

A number of optical imaging methods are available nowadays for 3D visualization of the growing vessel inside a tissue engineering bioreactor. The bioreactor example given in Figure 1 is monitored using an automated process of taking ultrasound cross sections non‐invasively. Scans are made at distinct positions during the rotation of the bioreactor housing and vascular graft facilitates a 3D visualization and the geometrical analysis of the growing tissue. At each distinct position, the probe scans the vascular graft along its full horizontal center axis to generate one cross section. The toolchain for the automated 3D visualization consists of the six principal steps and the software is implemented using the OpenCL framework.

A region of interest containing the vascular wall closer to the ultrasound probe is extracted from the automatically acquired cross‐sections. Binarization and morphological closing results in the segmentation of each cross section. Each segmented cross section is transformed into a cylindrical coordinate system, resulting in a rotational cross section model. A Cartesian grid is mapped onto the rotational cross section space and is used for the interpolation of the 3D graphic. Post‐processing removes interfering artifacts and computes the layer thickness and potentially present cavities. Crucial information on the vascular graft's wall strength and homogeneity, its possible weak points, resistance, and local distinctive features (e.g., plaque) are therefore obtained already before the end of the cultivation period. The provided geometric structure information also facilitates fluid dynamic simulations to further evaluate the mechanical characteristics of the vessel [29].

Other methods for 3D visualization, that contain a higher visual resolution exist in different formats: a Micro‐CT with a possibly cell deteriorating side effects of X‐rays [30], magnetic resonance imaging with its limited spatial resolution for single cell analysis [31]. Fourier Transform Infrared Spectroscopy (FTIR) is able to differentiate molecular scaffold compositions and may be usable to reflect cultivation‐depending transition processes. Confocal microscopy (CM) allows for optical sectioning, and reflectance‐CM based upon backscattered light was successfully adopted [32]. Multiphoton microscopy (MMP) based upon two‐photon excitation can be combined with second harmonic generation (SHG) for 3D‐imaging [33]. Another visualization feature with higher resolution than ultrasound imaging is given by optical coherence tomography (OCT). Time‐domain‐OCT for example uses low‐coherence near‐infrared light split in two arms (reference and sample). Here a beam splitter or fiber‐optic coupler produces 2D‐images of high resolution, only determined by the respective coherence length of the light source. These images can be transferred into 3D image reconstruction. Abide their high accuracy and resolution, methods such as OCT or MMP combined with SHG, however, have only a low penetration depth of up to 500 μm, and their use as monitoring tools in bioreactor system is only partially realized [34]. In addition, the hardware effort for all these techniques is enormous.

### 3.4 Monolayer generation on tubular scaffolds in a bioreactor

**FIGURE 2:**
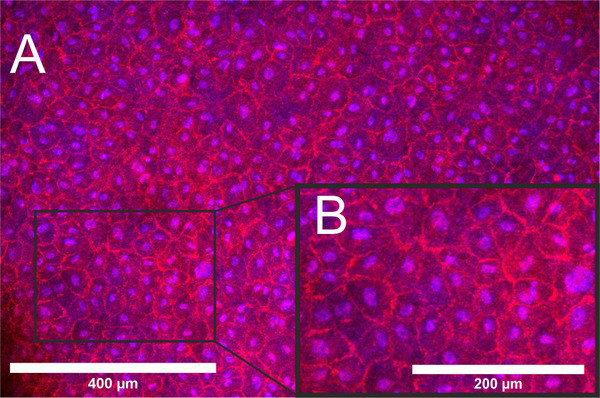
Endothelialization of a porcine vascular scaffold in a bioreactor system. Fluorescence staining of fixed human umbilical vein cells (HUVECs) on the inner surface of a porcine vessel after 7 days of cultivation in a bioreactor system under quasi‐static conditions (<0.5 dynes/cm^2^), cell nuclei blue‐stained with Hoechst 33342; cell limits red‐stained with VE‐cadherin. (A) Overview. (B) Detailed image; bars indicating the magnitude

To demonstrate the suitability of the bioreactor system depicted in Figure 1 for a real cultivation, Figure 2 presents an exemplary TEVG: a recellularized porcine vessel was seeded with human umbilical vein cells (HUVECs), and cultured for 7 days under laminar‐flow conditions with <0.5 dynes/cm^2^ ( = quasi‐static conditions).

Fluorescence staining revealed a confluent layer of endothelial cells on the inner surface of the vessel, which is crucial for subsequent vascular functionality. Dynamic cultivations with up to 20 dynes/per cm^2^ are still ahead, and in this context, others have successfully cultured fibrin‐decorated xenogeneic scaffolds with SMCs under a flow rate of up to 10 ml/min, and here a burst pressure of up to 200 mm Hg could be reached [35].

## 4 TISSUE ENGINEERING TRENDS IN VASCULAR SCAFFOLD DESIGN

### 4.1 Xenogeneic and allogenic scaffolds

Xenogeneic and allogeneic scaffolds were tested as possible natural scaffold materials. Decellularization concepts aim to avoid immunogenic reactions but also residual concentrations of cell‐toxic detergents in the recipient, and here an alternative concept for decellularization based upon caustic soda was realized by Eyre, Haverich et al. [36]. Dahan [14] produced a venous vessel from a decellularized porcine matrix seeded with endothelial and vascular muscle cells using dynamic cultivation in a bioreactor, and could show, that in particular, the use of vascular muscle cells contributed decisively to the mechanical stabilization of the vessel. However, according to Boyd [37], xenogeneic scaffolds frequently have to be removed due to inflammation. Olausson therefore successfully implanted a decellularized human but allogeneic vessel ‐ seeded with autologous cells‐ into a 10‐year‐old girl [38], but this remained a single study, and generally the use of human allogeneic scaffolds does not solve the intrinsic resource problem with lack of human vascular source material at this site. Consequently, other natural matrices are currently explored.

### 4.2 Natural materials

The review by Cordelle [39] describes silk scaffolds created by different methods (spinning, dipping, weaving, and electrospinning) and different surface modifications of silk. The use of fibronectin led to maximal cell adherence and heparin had a positive effect on later hemocompatibility. Small diameter vessels were also created using mulberry silk [40]. The graft resulting from colonization with porcine cells exhibited a burst pressure even higher than that of a native vessel. The silk as scaffold material showed an anti‐thrombogenic effect and proved to be favorable possibly due to a controlled degradation process over one year. Mechanical resilience was still 50% present two months after implantation.

Collagen is the most used natural polymer in vascular tissue engineering. As the main component of native extracellular matrix (ECM), it comprises properties such as low antigenicity, high biocompatibility, biodegradability, and is adhesive [41, 42]. Due to its poor mechanical properties and a fast degradation rate, modifying processes of collagen were tried, for example, using electrochemistry and electrospinning. Resulting scaffolds still imposed as very stiff and showed a disturbing high proliferative effect on cells. In contrast, vascular grafts out of mixtures of collagen with 25% synthetic PCL were found to be more flexible [43].

The more elastic fibrin gels (using fibrin formed out of fibrinogen after adding thrombin) are of benefit in vascular tissue engineering, since (i) it can be produced with the patient's own blood and (ii) it binds to critical proteins that direct cell fate, such as fibronectin and vascular epithelial growth factor (VEGF). One disadvantage could be that fibrin may interact with local coagulation cascades. Fibrin is mostly used as bio‐ink together with more stable scaffold materials [44], but was also manufactured as vascular graft, for example, by a molding technique [35]. Such fibrin vessels were often tested in the in vivo bioreactor approach with spontaneous cellularization by the living organism, but Swartz et al. implanted fibrin‐derived vascular grafts also with embedded endothelial cells with quite success in a sheep animal model. These grafts showed a bio‐elasticity comparable to coronary arteries [45].

Chitosan (CTS) is derived from chitin through its alkaline deacetylation, acts anti‐inflammatory and anti‐coagulative [46] and has a consistency comparable to glycosaminoglycans [47]. After careful processing using a new formulation of CTS with two different gelation processes (aqueous NaOH induced‐gelation and gaseous NH3‐induced gelation) and pH adjustment, the obtained gels with up to 10% CTS showed an increasing elastic module and average resistance and were considered as suitable material for vascular graft [48]. In vivo vascular implants out of CTS were randomly tested in rats and sheep. In addition, there are recent approaches with PCL‐electrospinned scaffolds decorated with CTS. Here CTS proved antibacterial and antithrombotic properties [49].

Elastin is a natural elastic polymer that occurs in the natural wall of arteries. Nguyen et al tested a combination of elastin with collagen in seeding experiments with regard to a potential vascular graft. The component elastin had an advantageous effect on ECM organization and contractility of seeded SMCs [50], and vascular constructs including polyurethane and elastin were already tested in vivo in rats [51].

Altogether, the natural materials presented here have excellent molecular properties for optimal cell growth, and some of them act as anti‐thrombotic agents. However, they are mostly used – except of silk – as decorating and cell adhesive material together with other, more stable materials in vascular tissue engineering, and were not consequently followed as single‐component‐vascular graft for patients up to now.

### 4.3 Biodegradable polymers

Scaffolds can also be produced out of biodegradable polymers. Here the idea is, that in parallel to the degradation process of the polymer, the seeded cells overtake the regimen and produce natural extracellular matrix and finally form a tissue layer. Currently, many different materials are available that can be considered as scaffold materials for a vascular prosthesis´ scaffold. PGA as an established polymer has a high melting point >200 °C, and high tensile strength (12.5 GPa) and was tested as vascular scaffold already 20 years ago [52], but exhibits a rapid decrease in mechanical properties due to its very rapid degradation. In addition, the release of the decomposition product glycolic acid–although degradable by cells via the citrate cycle–produces local inflammation. The PLA‐derivatives poly(L‐lactic acid) (PLLA) and poly(D‐lactic acid) (PDLA) have been extensively studied, and recently PLLA blended with poly‐caprolactone (PCL) was tested after decoration with heparin or VEGF in animal models with quite success [53]. The co‐polymer poly(lactic‐co‐glycolic acid (PLGA, composed of the L‐ and D, L‐lactide forms) is the most studied degradable polymer for biomedical applications due to ease of commercial production and has also been used for scaffolds. PLGA showed good cell adhesion and proliferation properties. Various methods were applied for shortening degradation times such as modifications or irradiation. In vivo, however, PLLA still could not be completely absorbed even after months [54]. Wang et al [55] successfully tested a scaffold design in which PCL was reinforced by a mesh of polyethylene‐terephthalat (PET). The tubular shape was created by clamping on a cylinder and the micro‐structuring and mechanical strength was comparable to that of expanded polytetrafluoroethylene (ePTFE), which is widely used as a conventional vascular prosthesis material. Daum developed scaffolds made of polyurethane (PU) which, after surface modification with fibronectin/decorin, were seeded with vascular cells (isolated from a vessel) [56]. The most degradable polymers, suitable for colonization of cells on the surface, have a very long degradation time, however, and it was observed, that they therefore did not fully integrate into the growing vascular graft. Furthermore, some of these polymers present as an impermeable barrier for cells with the consequence of necrosis through insufficient medium supply. A recent review carefully rates diverse biodegradable polymers concerning their degradation properties and comes to the following conclusion [57]: PGA and its copolymers, such as PLGA, degrade too rapidly and their tensile strength decreases by half within two weeks. PLLA degrades too slowly in a period of up to 6 years for maximum resorption. Lactide‐ε‐caprolactone copolymers (LA‐CL cop) were given preference because of this unsatisfactory resorption property of PGA and PLLA. The degradation rates of well evaluated synthetic biodegradable polymers decrease in the following order: PGA∼PLGA > Poly‐DL Lactic Acid (PDLLA) > PLLA > PCL. It could therefore be a good concept to combine a mechanically stable polymer such as PCL with a faster degrading polymer for surface structuring in the same scaffold. Such a polymer is, for example, represented by polydioxanone (PDO), that is very suitable for vascular cell seeding, and shall be discussed in the following [58].

### 4.4 Polydioxanone as a new material for vascular scaffold design

**FIGURE 3:**
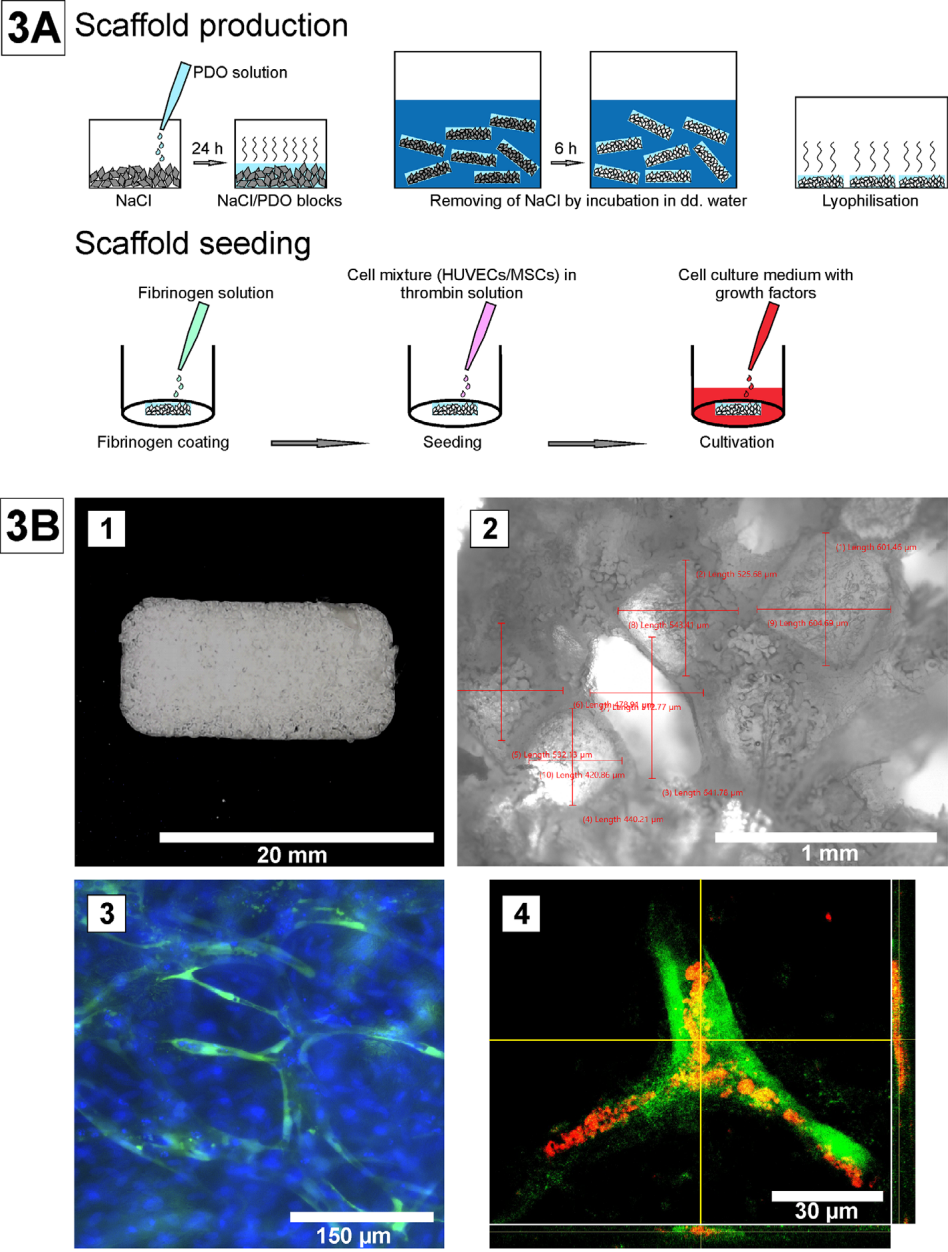
Capillary network on a macroporous PDO scaffold, generated by porogen‐leaching. (A) Poly‐p‐dioxanone scaffolds were produced by dissolving PDO in hexafluoro‐2‐propanol using a porogen leaching process with NaCl crystals as porogen [58, 81]. (B) 1–4: 1) Macroporous PDO‐scaffold, 2) Dimensions of pores, 3) Tubule‐like‐structures out of GFP‐labeled HUVECs (green) and MSCs; cell nuclei stained with Hoechst 33342 (blue), 4) Lumen‐verification using a dextran assay (GFP‐HUVECs, green; dextran, red) [58]

Of note, PDO is a colorless semi‐crystalline polymer with a melting point of 180–190°C. PDO ‐ considered to have a fast to moderate degradation time (∼3–6 months) ‐ is not very deformable, but flexible and is well established as a suture material for 30 years as monofilament PDS. PDO has already been tested in combination with adipogenous tissue derived mesenchymal stem cells (AD‐MSCs) [59] and supported the differentiation of stem cells into bone and adipose tissue. PDO filaments can be used to produce macroporous scaffold by a porogen leaching method e. g. making use of NaCl as porogen (Figure 3A) [58]. These structured scaffolds can be integrated for cultivation of microvessels–as present as “vasa vasorum” in the vascular wall–to maintain nutrient supply for thicker arterial walls such as in the aorta.

**FIGURE 4:**
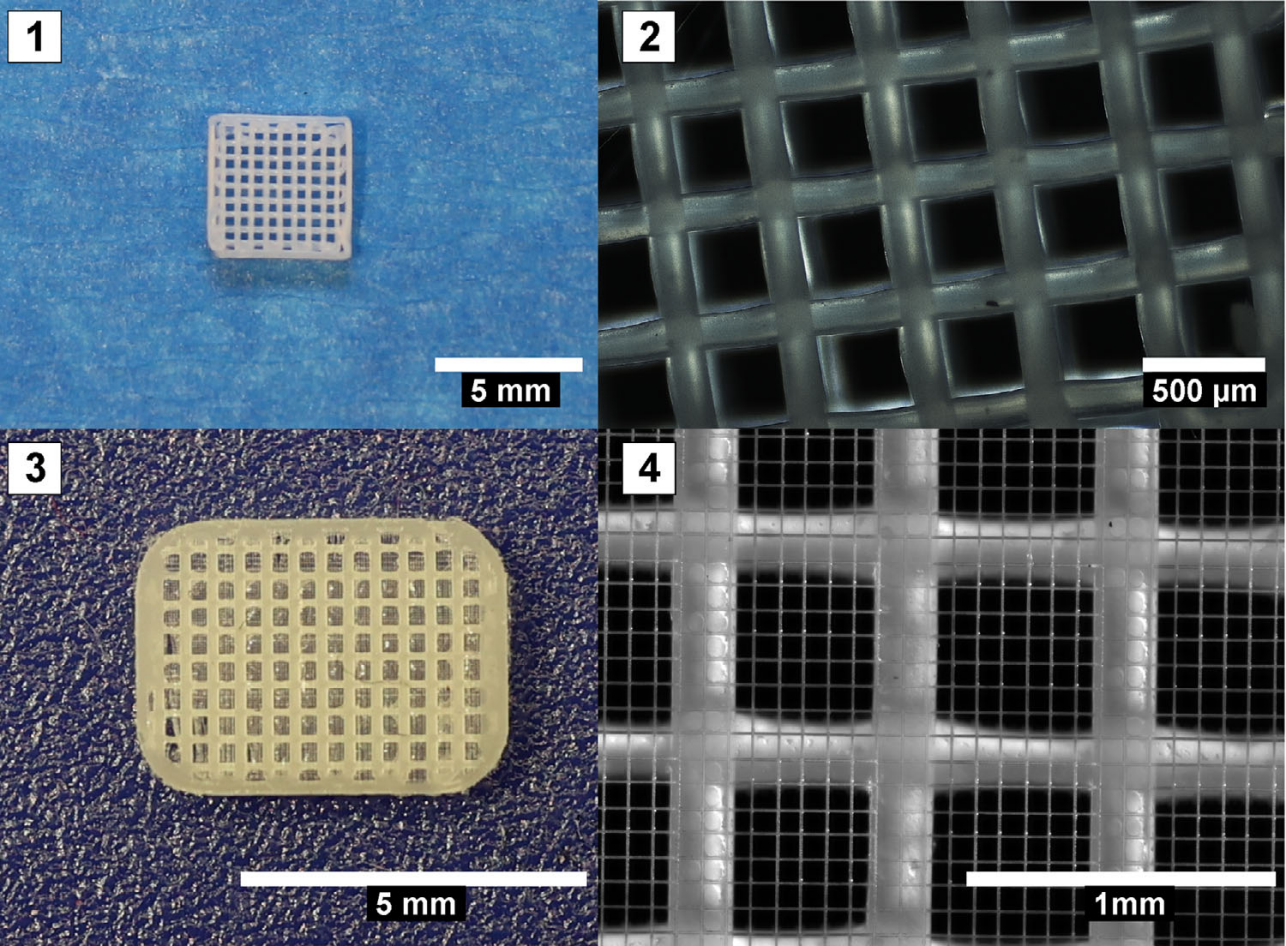
3D printing to generate vascular scaffold microstructures. Pictures A and B show examples of 3D printed meshes with millipores (0.1–1 mm) for optimal cell growth, printed with a 0.1 mm nozzle at 140°C by a extrusion bioprinter (Cell Ink Co., Sweden). Pictures C and D show a combination of extrusion printed meshes and MEW‐generated micrometer pores (Bioprinter by RegenHu Co., Switzerland; unpublished results)

As depicted in the schematic of Figure 3A, PDO was combined with fibrin to increase cell adhesion during cultivation (Figure 3A). AD‐MSCs [60] are autologous cells and act as perivascular cells enabling optimal ingrowth of the endothelial cells (EC) into the pores (Figures 1, 3, 1 and 2). In addition, human AD‐MSCs promote tubular network formation of EC in suitable medium, supplemented with growth factors, for example, components such as VEGF, hydrocortisone, bFGF and others (Figure 3B, 3 and 4) [61]. PDO here plays a role to provoke capillary structure building, which is crucial as integrative element to nourish thicker vascular walls. PDO was also used in electrospinning approaches as vascular wall material [62]. The use of PDO in combination with the natural polymer fibrinogen seems promising also in 3D printing.

### 4.5 Electrospinning as method for scaffold design

The method of electrospinning nanofibers on a cylinder has also long been explored for development of tubular vascular scaffolds. Electrospun tubes were wrapped in a thicker polymer coil in order to improve the mechanical properties of these vascular scaffolds. Constructs seeded for example with MSCs were tested in a rabbit model and showed long patency throughout implantation [63]. The disadvantage of electrospinning – however – may be, that this method is highly complex, leads to chaotic structures with diverging quality. Some groups combined electrospinning with 3D printing for better results. Combined materials such as chitosan (CTS) and PCL, as well as blended CTS/PCL or heparin‐eluding PLLA for better anti‐thrombogenicity were used [64].

### 4.6 3D printing for generation of vascular scaffolds

3D printing represents a new approach for matrix and scaffold fabrication and belongs to the class of additive manufacturing processes in which, in contrast to the classic subtractive processes (e.g., milling), the desired object is produced by applying material layer by layer. Numerous different printing processes have developed from the various fields of application, the variety of materials processed and other general conditions such as requirements for accuracy or surface finish of the manufactured parts. Regardless of the 3D printing process, the workflow generally comprises three main steps: (i) The desired geometry is created with the help of computer‐aided processes, usually using CAD software before (ii) being processed by specialized slicer software, which breaks down the geometry into individual layers (“slicing”) and generates printer‐specific commands from each layer. The command set is processed by the 3D printer, and (iii) the desired geometry is manufactured by selective material deposition [65]. 3D printing offers the possibility to arrange support materials, cells or active agents in a geometrically defined structure, and thus 3D printing drives the development of three‐dimensional cell culture processes as in tissue engineering. From a technical point of view, 3D printing processes often differ only slightly from the industrial model. In most cases, only an adaptation of the material deposition techniques to the materials used in 3D printing, as well as a development of these very materials, for example, of hydrogels, biocompatible plastics, etc., takes place. The geometry creation and workflow are essentially similar to technical 3D printing. Increasingly, however, there is an awareness of the need for adapted CAD/computer‐aided manufacturing (CAM) and slicing software, and this is giving rise to corresponding research and development activities. The incorporation of medical imaging for patient‐specific scaffold development is also increasingly being explored in this context and must be optimized in the future [66]. Creation of a microporous tubular structure as vascular scaffold, with a suitable diffusion barrier for seeded cells out of diverse synthetic or natural polymers, therefore appears feasible using 3D printing.

Porosity generated by 3D printing with significantly smaller pore diameter of scaffolds supports cell adhesion and can be even more enhanced by staggering the layers and using lattice patterns as well as hexagonal and gyroid patterns. Polymers used in the context of 3D printing can be processed by digital light printing (DLP). One example is poly (propylene fumarate) (PPF), a biodegradable polyester. This compound is a UV resin, solved in diethyl fumarate, and is activatable via bisacrylphosphrine oxide as photoinitiator for crosslinking [61]. PPF‐scaffolds were produced by DLP with an inner diameter of 1 mm and a wall thickness of 150 μm. PPF‐scaffolds showed long‐term mechanical stability and sewability and a high tensile strength of 1.48 MPa and elastic modulus comparable to native femoral arteries and saphenous veins. These grafts were tested in mice, were remodeled and endothelialized, the only disadvantage was an extended inflammation [67]. Polyurethane (PU) as a non‐bioresorbable polymer with a high mechanical strength was also processed by DLP. Tubular constructs derived from PU showed high porosity and interconnectivity as well as a high suture tear resistance. Light‐inducable polytetrahydrofuran diacrylate (PTHF‐DA) was successfully processed using stereolithography to generate filigree bifurcating tubes with small diameters of 2 mm with an elastic modulus of 6 MPa [68]. PCL again represents a biodegradable polymer with a relatively long‐during degradation time over several years. Nevertheless, many 3D printing approaches were based on PCL so far. PCL is an FDA approved biodegradable polyester with a low stiffness and was often combined with other polymers [69] to create a structure with a lumen or in a procedure combining 3D printing with electrospinning.

Summarizing, 3D printing offers the benefit of producing scaffolds fast and reproducible with a highly defined structure. The challenge for 3D printing with regard to vascular grafts is to produce a seamless tubular structure with high mechanical elasticity. The following Figure 4 shows 3D printed meshes out of PDO as a macro‐structured scaffold for cell seeding, (A–C) and melt‐electro‐written PCL fibers in micrometer‐scale reinforced with a printed mesh (D).

Future developments in 3D printing must also comprise concepts for printing multilayered vascular wall structures, suitable as blood vessel scaffolds. This and the delicacy of the resulting process vote for an exploration of the method of Melt Electro‐Spinning and Writing (MESW) as an attractive new method in 3D printing. The Melt Electro‐Writing (MEW) process is attracting special attention in the field of bioprinting, as this method allows very thin fibers with a fiber diameter of typically 2–50 μm that can be deposited in defined structures [70].

## 5 SUITABLE CELLS SOURCES AND CELL CONDITIONING

### 5.1 Suitable cell sources

As mentioned earlier, not only the antithrombotic surface design of suitable scaffolds, but also mechanical resilience is an important issue in a vascular graft. This led to a number of experimental approaches in which, in addition to surface modifications, different cell sources were used for colonization and even multicellular wall structures could be created.

Heine and Haverich [71] described the production of a blood vessel with three layers. It consisted of i) an intima with (non‐allogeneic) endothelial cells (and here the group used human umbilical vein cells; HUVECs), ii) a media with vascular SMCs , iii) an outer layer corresponding to a native adventitia with vasa vasorum produced after co‐cultivation with mesenchymal stem cells and HUVECs. The vascular muscle cells also emerged from the stem cells under the influence of Transforming Growth Factor‐β (TGF‐ β) [72]. Although such approaches are an amazing step into the direction of artificial bio‐hybrid vascular grafts, generally, isolation out of human aortal vascular structures would not solve the resource problem consisting in the low availability of human material for bypasses. This is especially true for older patients as potential donors that often suffer from high comorbidity.

Xenogeneic cell isolation protocols were successfully explored [73] but as discussed earlier, their immunogenic risk hinders from use in a vascular implant for patients. Ghorbel et al therefore stepped forward and used vascular SMCs developed out of human umbilical cord blood stem cells under the influence of TGF‐β [72] on porcine scaffolds. Spontaneous endothelialization was then awaited in small pigs in an in vivo long‐term experiment, the vessels were assessed after 5 months and showed bi‐layering (intima and media). To further minimize the immunogenicity, autologous human smooth muscle outgrowth cells (SOCs) were suggested for use in TEVGs. These cells have already proven suitability in intravascular gene therapy. An elegant method to obtain these cells is to develop SOCs out of differentiating progenitor cells from peripheral blood [74]. SOCs exhibit a morphology similar to that of SMCs with a mountain‐and‐valley morphology. SMCs are recognizable by specific markers such as calponin, α‐SMA, and SM myosin heavy chain, but also provide the markers VEGF, elastin, matrix Gla protein (MGP), and smooth muscle protein 22α (SM22α) and integrin α5β1. Xie et al. showed, that SOCs can be used for colonization of a scaffold. After three weeks of cultivation under low flow conditions (<0.5 dynes/cm^2^) on silk‐fibroin‐modified poly(3‐hydroxybutyrate‐co‐3‐hydroxyhexanoate (SF‐PHBHHx), a confluent structure developed in the form of lamellae [75].

The problem with autologous cells is that they are needed in high numbers for endothelialization of suitable scaffolds. A cell isolation method for obtaining endothelial progenitor cells (EPCs) from peripheral blood was earlier established and recently optimized.

**FIGURE 5:**
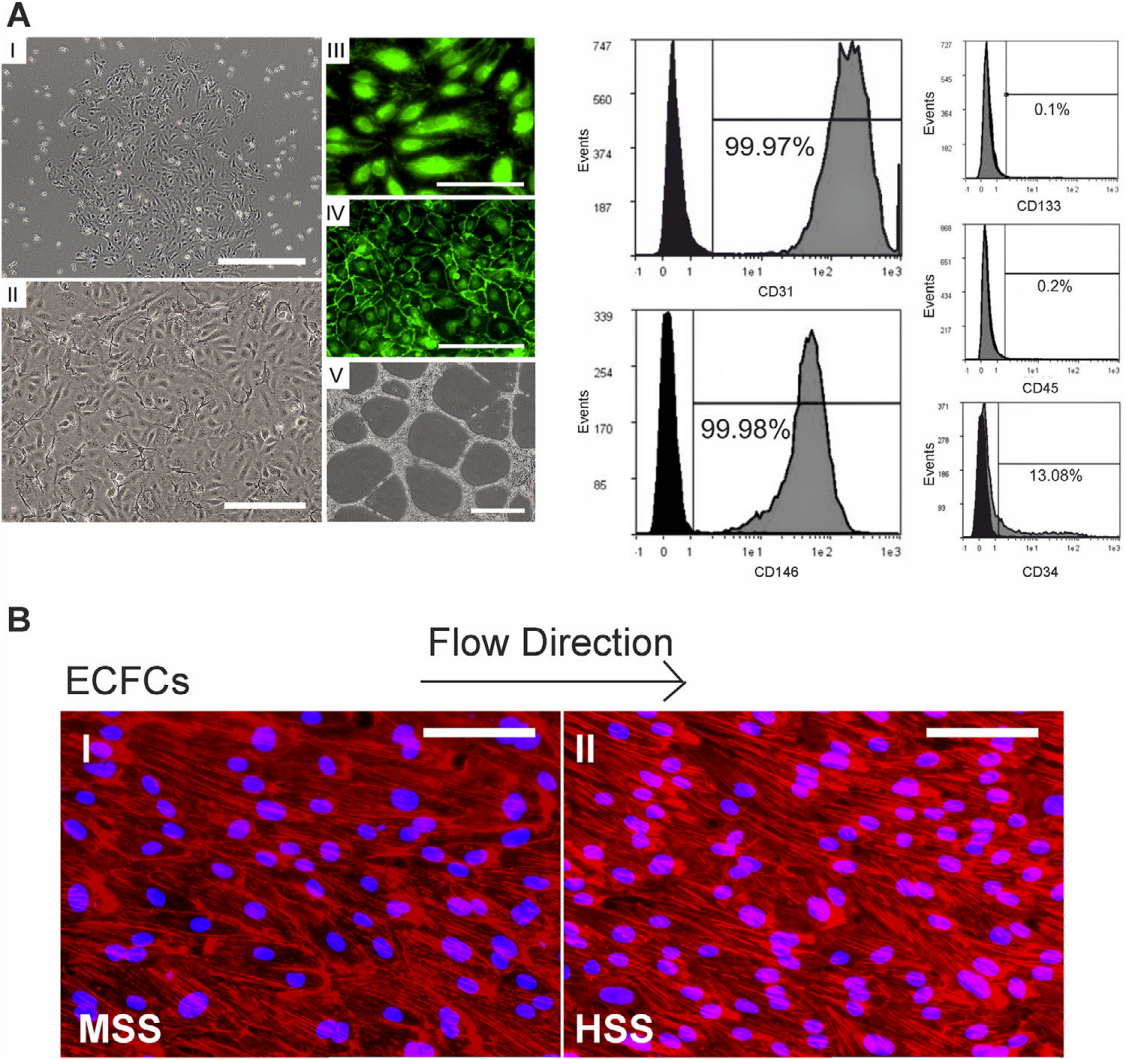
Characterization and results of dynamic cultivation of endothelial colony forming cells (ECFCs), isolated from peripheral blood. (A) (Left side) ECFCs in I‐II with typical cobblestone morphology after up to 28 days, green immunofluorescence staining of vWF (III) and VE‐cadherin (IV), and capillary‐like structures in the angiogenesis assay (V), with typical antigen patterns in flow cytometry such as CD31+, CD146+, CD34+, CD133‐, CD45‐(right side). Scale of figures: I: 500 μm, II: 250 μm, III: 200 μm, IV: 100 μm, V: 1 mm. (B) Enforced cell orientation of ECFCs, detectable by staining of F‐actin filaments after pulsatile (1 Hz) cultivation with high shear stress (II) as compared to medium shear stress (I); ECFCs (MSS: medium shear stress = 5 dynes/cm^2^; HSS = high shear stress of 20 dynes/cm^2^; Scale in all images: 100 μm); adopted from [8]

Mononuclear cells are selected during cultivation and characterized as endothelial cell forming cells (ECFCs) as a subset of EPCs by flow‐cytometric analyses [8] These cells represent premature endothelial cells with typical cobblestone formation and expression of endothelial markers (VE‐cadherin/vWF) with preserved angiogenic capacity as shown by angiogenesis assays on a three‐dimensional matrix (Figure 5A).

As an alternative solution to the concept of isolating autologous cells with ex vivo expansion, human leucocyte antigen (HLA)‐type‐matched human inducible pluripotent stem cells (hiPS) may also be used. In parallel, HLA‐gene edited hiPS with reduced HLA‐molecules I and II expression are being developed [76]. These iPS – however – have to be maturated to get to the phenotype of the target cells, for example, endothelial or smooth muscle‐cell‐like, which is complex. As mentioned earlier, this process does not always lead to a maturation grade equivalent to autologous differentiated cells, so for example, ECM components appeared less robust [7] and present a comparably decreased mechanical strength as part of hiPS‐TEVGs [15]. Consequently, the differentiation protocols for the use of hiPS still have to be optimized.

### 5.2 Conditioning of cells

For conditioning of either endothelial or SMCs, dynamic cultivation in a suitable bioreactor is important and supports the differentiation process of immature progenitor cells into mature vascular cells. It was for example beneficial for the differentiation of mesenchymal stem cells to smooth‐muscle‐cell‐like cells on suitable biodegradable scaffolds (made of PCL/PLA). Dynamic cultivation here may comprise not only laminar flow but also pulsatile flow conditioning, and may thus have anti‐thrombogenic and anti‐inflammatory effects promoting the generation of a vascular graft not endangered by fast thrombogenicity after implantation [77]. How deep‐going the impact of pulse on vasoreactivity of cells is can be shown by Tosun et al.. In a perfusion bioreactor, temporally variable pulse frequencies versus constant pulse were applied on decellularized umbilical cord scaffolds, seeded with vascular SMCs. Whereas the SMC exposed to constant pulse expressed a contractile phenotype, the variable pulse frequencies drove cells to a synthetic state with continued cell proliferation, increased tensile strength and stiffness as well as diminished vasoactivity [78]. Nitric oxide is considered as a “marker of success” indicating vasoreactivity in mature endothelial cells. In this context, Uzurski described umbilical cord scaffolds with HUVECs that successfully generated nitric oxide when exposed to undulating pulsing in a bioreactor [79]. Von Hafften et al showed, that cyclic pulsatile flow had an anti‐inflammatory effect on macrophages and fibroblasts invading a successfully endothelialized "electrospun" PCL/N,N′‐bis(trimethylsilyl)urea‐scaffold. Future dynamic cultivation concepts should therefore take also variable pulsatile flow into account [80].

We report an approach for dynamic cell culturing for ECFCs. ECFCs exhibit significant changes in the axial ratio after both laminar (LC) and pulsatile (PC) shear stress cultivation, whereas for endothelial cells from a cell line such as HUVECs a significant change is only observed after pulsatile flow (Figure 5B). During dynamic cultivation, ECFCs not only build a highly interconnected monolayer but also an antithrombogenic gene profile [8]. All these alterations are more pronounced than in endothelial cells from a cell line such as human umbilical venous endothelial cells (HUVECs), favoring the idea, that ECFCs have not only the advantage of not being rejected due to immunogenicity but are also more responsive to culture conditioning than HUVECs.

## 6 CONCLUSIONS

Bio‐artificial grafts, that have already reached the level of clinical trials, were mainly composed of immune‐neutral components to prevent thrombosis. Some of them showed a mechanical weakness and developed stenosis. Cell conditioning and graft maturation in a bioreactor therefore appears advantageous.

Suitable bioreactor systems, equipped with multi‐modular functions, are the basis for the successful production process of a vascular graft and seem in tangible proximity. A bioreactor system can act as a test setup providing physiological conditions, thus simulating the human body during vasculogenesis. Various harsh conditions for electronic elements have to be considered, and corresponding suitable solutions have to be realized. Dynamic cultivation is able to produce mechanically stable endothelial monolayers on the synthetic scaffold and trains the cells to expose an anti‐thrombogenic character. Ultrasound monitoring and 3D visualization of the growing graft in the bioreactor containment is practical, but has a comparably low image resolution. In contrast, alternative methods such as OCT, MMP, and SHG with high resolution have a minor penetration depth and may only allow surface studies of the vascular grafts and are not yet integrable in bioreactor systems due to their enormous apparative expenditure up to now.

Among the diverse materials used for vascular tissue engineering, natural gelatinous materials such as fibrin, chitosan, and others have not yet found their way as scaffold‐free vessels resilient enough for clinical use. Silk by contrast is a very attractive material especially for manufacturing with electrospinning. There are many suitable, biocompatible materials such as biodegradable polymers that were tested with cells so far. Here exists the vision, that cell matrix production will overtake the mechanical strength of the earlier scaffold. For their successful use as part of a vascular graft, degradation properties will be of major importance. Some of these polymers were processed as electrospun vessels, and most of them were decorated with adhesive additives for optimal cell growth and increased anti‐thrombogenic or antimicrobial properties. A new trend is to generate vascular 3D printed tubes with microstructured surface or as macroporous scaffolds for capillary structures. Among the huge number of varying polymers tested so far, PCL as outer support, PDO as inner layer with its medium degradation time and human fibrin with adhesive properties may be a promising new combination of a natural and synthetic polymer to build up a vascular scaffold. Processing different biodegradable polymers by 3D printing in form combined extrusion printing and melt electrospinning writing represents a new attractive technology to generate such multi‐material pre‐designed, microstructured scaffolds. Of course, the resulting biomechanical stability must be extensively tested, but some 3D printed scaffolds have already shown a sufficient quality.

Recruiting a cell number high enough for seeding vascular tissue engineered scaffolds, but with a low immunogenic profile, remains a problem to be solved. Xenogeneic and allogenic cell sources impose the risk of immunological identification and following fast immunological clearing reactions. In contrast and besides promising results concerning iPS, there are possibilities to isolate autologous endothelial progenitor cells e. g. out of blood and expand them. Furthermore, blood isolated smooth muscle outgrowth cells, which are differentiated to target cells in a distinct milieu, show great potential to be used as an autologous substitute for SMCs.

Based on all these promising new approaches, standardized procedures for generation of vascular grafts are envisioned. Results should withstand durability tests to act as new and powerful replacement vessels especially for elderly or comorbid patients without alternatives. 3D printing here seems to be a highly sophisticated supporting technology in this context.

## CONFLICT OF INTEREST

The authors declare that there are no conflicts of interest.

## ETHICS STATEMENT

There were no ethical or legal concerns about the use of liposection material for isolation of adipose tissue derived human mesenchymal stem cells shown in some of the exemplary results presented here. Liposection material was obtained after operations in the Dept. of Plastic and Reconstructive Surgery of the Medical School Hannover (MHH), and this was audited on behalf of the ethics committee of the MHH (reference number 3475‐2017).

## Data Availability

The data that support the findings of this review are available from the corresponding author upon reasonable request.
